# Accessibility of care plan information from previous treatment setting in palliative care unit: A qualitative study

**DOI:** 10.1002/nop2.1315

**Published:** 2022-08-21

**Authors:** Anne Kuusisto, Kaija Saranto, Päivi Korhonen, Elina Haavisto

**Affiliations:** ^1^ Department of Nursing Science University of Turku Finland Turku Finland; ^2^ Satakunta Hospital District Pori Finland; ^3^ Department of Health and Social Management University of Eastern Finland Kuopio Finland; ^4^ Department of General Practice, Turku University Hospital University of Turku Turku Finland; ^5^ Department of Health Sciences Tampere University Tampere Finland

**Keywords:** advance care planning (mesh), care planning, care plan, care planning, communication, continuity of patient care, electronic health records, health information systems, information, nurses, palliative care

## Abstract

**Aim:**

To describe accessibility of care plan information from patients' previous treatment setting in palliative care.

**Design:**

A qualitative descriptive study.

**Methods:**

A total of 33 nurses, social workers and physicians were interviewed. Data were analysed by deductive and inductive content analysis. The Fit between Individuals, Task and Technology (FITT) framework was used as a deductive analysis framework.

**Results:**

Individual‐task Fit was described in relation to professional‐specific care plan information in palliative care and use of time to obtain care plan information. Individual‐technology Fit was described in relation to health informatics competencies and HIS usability. Task‐technology Fit was described in relation to interoperability between care settings and healthcare providers and lack of interoperability between care settings and healthcare providers.

**Relevance to clinical practice:**

The study confirms the need to review the HIS as a whole from a holistic and patient‐oriented perspective to ensure the continuity of palliative care.

## 1 INTRODUCTION

When patients move from one institution to another, it is essential that healthcare professionals have access to care plan information on how patients' care will continue. Health information sharing between different organizations and professionals is particularly important in palliative care where access to patient information is an enabler to serve appropriate end‐of‐life care (Patterson et al., 2019). For example, absence of proactive care planning has been one of the most common barriers to optimal palliative care (Patterson et al., 2019; Uitdehaag et al., 2018).

Health information exchange is dependent on the legal, economic, cultural and political context of health care and is related to national activities and service system structures. Different countries vary extensively in their level of accessibility of information in electronic format suited for health information exchange on national, regional and local levels. (Payne et al., 2019.) Despite technological advances (HL7, 2022; Payne et al., 2019; Sockolow et al., 2020), access to care plans remains a challenge, threatening the continuity of palliative care (Hopkins et al., 2017; Uitdehaag et al., 2018). To ensure continuity of care, it is important to know if patients' care plan information from the previous treatment setting is accessible in the palliative care unit.

### 1.1 Background

As patients receiving palliative care move between healthcare settings, their care plans should be accessible to stakeholders to ensure continuity of care (HL7, 2022; Uitdehaag et al., 2018). The patient care planning literature includes a variety of concepts, studies and interventions (Keenan et al., 2008). According to a scoping review, care plan information in palliative care consists of care plans and its special form of advance care planning (ACP) for end‐of‐life issues. The use of the terms is still rather unclear among professionals (Kuusisto et al., 2020).

A care plan is an individualized document (American Medical Association, 2016; Casotto et al., 2017) and includes ACP (Vaitiekunas et al., 2021). If accessible, care plans have been important information sources (Østensen et al., 2020), improved transition to palliative care, have had a beneficial effect on patient care outcomes such as quality of life (Hopkins et al., 2017) and shown a protective effect in healthcare setting transitions (Casotto et al., 2017). A recent scoping review shows that nurses can ease patients' transitions between care settings with thoroughly discussed healthcare decisions and documentation of end‐of‐life wishes (Fliedner et al., 2021). Ideally, each patient has ACP, so nurses can work without consultation with a physician, e.g. on weekends or at night (Uitdehaag et al., 2018).

In medicine, care plan is a template built into health information systems (HISs). It includes, e.g. problem list, medication list, diagnosis, prognosis, co‐morbidities, the patient's goals of care, treatment, management of side effects and ACP. In addition to the patient, it is used by other professional groups such as nurses (Vaitiekunas et al., 2021.) In nursing, a nursing care plan encompasses problem, goal and interventions (Østensen et al., 2020). Performing care plans and use of data are core competencies in Health Informatics for nurses and should be taught about them and other healthcare professionals (Hübner et al., 2018).

The advent of technology should in theory help with documentation and transfer of information (Haux, 2006). But previous studies show that the benefits of technological advances, such as easy access of care plans (Hopkins et al., 2017) and interoperability between HIS, have not yet been reached (Taggart et al., 2021). In Australia, after introduction of a care plan for patients with cancer, less than half of the nurses thought that they were easy to access (Hopkins et al., 2017). Later, no two‐way electronic care plans were accessible nationally and locally (Taggart et al., 2021). In the USA, longitudinal care plans existed in one setting, but were not shared across settings. The most common formats for care plan communication were paper and fax (Dykes et al., 2014). One challenge is that the information needs of care plan recipients are different from those of information producers (Østensen et al., 2020) and differ depending on the professional group (Haavisto et al., 2020). Recently, Sockolow et al. (2020) highlighted the need for continuity of care standards for interoperability in HIS to support information sharing. A new scoping review showed that standardized terminologies have potential to improve documentation and interoperability across settings, for example (Fennelly et al., 2021).

**FIGURE 1:**
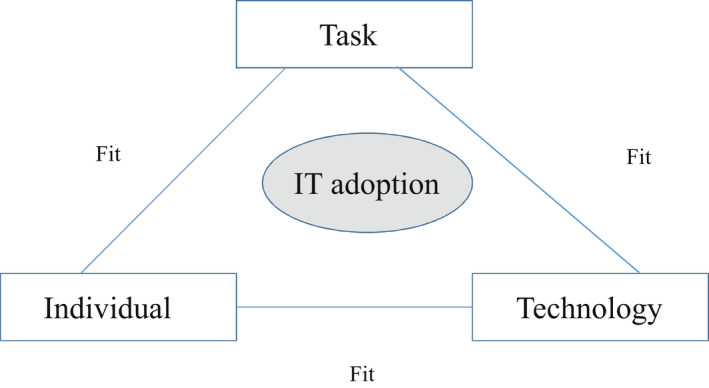
The FITT framework; IT adoption depends on the fit between the individual, task and technology (Ammenwerth et al., 2006)

Health care represents a complex socio‐technical system where people and technologies interact. The amount of data generated, the number of individuals requiring access and the number of locations where data are stored increase all the time (Whetton, 2005). In health care, HIS approval means optimal interaction (fit) of *individual*, *task* and *technology*. The “Fit between Individuals, Task and Technology” (FITT) framework specifies these socio‐technical elements that effect the HIS approval. The features of the *individuals* include, e.g. computer enthusiasm or anxiety. The features of the clinical *tasks* contain, e.g. work processes or organizational factors. The features of the *technology* involve, e.g. usability or functionality (Figure 1). The framework points out that for the HIS approval, the fit between different features is more important than the individual features themselves. For example, computer enthusiasm of the individuals is not adequate for the success of an HIS implementation—preferably, they must fit the HIS functionality (Ammenwerth et al., 2006).

Taken together, in health care, the majority of systems consist of living (people) and non‐living (information, technology) components. They are socio‐technical systems. Issues for health informatics include privacy and security, integrating technology into clinical workflows, usability and identifying the optimum information tools for each clinical setting and professional. When designing socio‐technical systems, it is important to understand how people and technology interact. (Whetton, 2005.)

## 2 THE STUDY

### 2.1 Aim

The aim of this study is to describe the accessibility of care plan information from patients' previous treatment setting in the palliative care unit. This study expands our understanding of information transferability by describing how health information becomes accessible when patients transferred from one setting to another. This study is part of a larger study designed to improve continuity of care. The extensive data have been gathered from diverse healthcare professional group, but also from patients and family members.

### 2.2 Design

A qualitative descriptive study was conducted. Qualitative descriptive design is especially flexible to receiving straight and largely straightforward answers to questions of special relevance, e.g. to healthcare professionals. (Sandelowski, 2000). Data were analysed by deductive and inductive content analysis. A deductive analysis framework (FITT) was used (Ammenwerth et al., 2006). The COnsolidated criteria for REporting Qualitative research (COREQ) guidelines (Tong et al., 2007) were followed (Appendix S1).

### 2.3 Sample

The purposive sampling strategy was applied to select interviewees that could provide appropriate and well‐saturated data needed to achieve the study aims (Polit & Beck, 2017). A desired sample of best informants by group were nursing staff (group interviews; *N* = 6; 4–7 nurses/group) and physicians (individual interviews; *N* = 5 physicians) who were the largest professionals groups in palliative care. In addition, the view of the social sector was wanted (individual interviews; *N* = 5 social workers). The study was conducted in three hospital districts in Finland because of their geographical representativeness. Almost half (42%) of the Finnish population lived in these areas. The vast majority of the palliative special level wards located also in these areas.

Registered nurses, practical nurses, social workers and physicians were recruited to be interviewed by the participating organizations. The inclusion criteria were volunteering to participate and working in a palliative care unit in immediate patient care. Each unit had appointed a contact person to coordinate the research who took care of the recruitment of the interviewees and issuance of the research bulletin and consent form and acted as a liaison for the researchers. The researcher arranged the interview time and place. Overall, 33 healthcare professionals participated in the study. Their ages varied from 19–62 years (mean age 46 years). They had work experience in health care for <2 years to 37 years and from <1 year to 19 years (mean 6 years) in palliative care.

### 2.4 Data collection

The data were gathered via focus group and individual interviews as planned in three hospital districts with one outpatient clinic and five wards in spring and autumn 2019. Practical nurses and registered nurses participated in focus group interviews (Freeman, 2006). Social workers and physicians participated in individual interviews. Individual interviews were a method because no focus groups including social workers and physicians would else have been reached. Instead of individual interview, one couple interview of registered nurses was made due to the operation of the ward and another at the ask of the physicians.

Guideline‐based interviews (McGrath et al., 2019) were by occupational group face‐to‐face sessions in the workplace, one by telephone and another in a separate space without interference from other participants. (Table 1). Professionals were asked to describe (when new patients come to their unit) the accessibility of care plans from patients' previous care setting. The audio‐recorded interviews lasted between 29–173 min (mean 58 min).

**TABLE 1:** Description of data collection and the healthcare professionals involved (*N* = 33)

Interview type	Professional group	Number of professionals
Focus group FGPN1	Practical nurse	5
Focus group FGN1	Registered nurse	6
Focus group FGN2	Registered nurse	4
Focus group FGN3	Registered nurse	3
Focus group FGN4	Registered nurse	3
Couple interview CIN1	Registered nurse	2
Couple interview CIP1	Physician	2
Individual interview P1–P3^a^	Physician	3
Individual interview S1–S5	Social worker	5

^a^ “P3” = telephone interview.

### 2.5 Ethics

The study followed good scientific practice and research ethics guidelines (World Medical Association, 2022). Research permits were obtained from organizations and Research Ethics Committee approval from University Research Ethics Committee (15/2019). The interviewees gave their consent to participate after they were informed about the study procedures, voluntary and confidential participation. The researchers who collected the data were persons from outside the palliative care unit; no relationship was thus established prior to study commencement. Before the interviews, the interviewers introduced themselves.

### 2.6 Data analysis

The data were analysed with content analysis using combined deductive and inductive approaches (Polit & Beck, 2017). Before analysis, a structure of deductive analysis for the theme accessibility of care plans from patients' previous care setting was defined based on the FITT structure (Ammenwerth et al., 2006; Sheehan et al., 2012). In this study, Individual‐task Fit, Individual‐technology Fit and Task‐technology Fit from the FITT framework serve as categories that unite the theme accessibility of care plan information from patients' previous care setting. “Individual” represents healthcare professionals (registered nurses, practical nurses, social workers and physicians) who take care of patients in the palliative care unit according to their professional skills and requirements. “Task” covers the set of clinical duties and work processes concerning access to care plan information from patients' previous treatment setting by healthcare professionals with defined responsibilities that are supported by the given technology. “Technology” stands for the interaction of all tools (e.g. computer and paper‐based tools, phone) needed to access care plan information from patients' previous treatment setting used by the healthcare professionals.

First, the data were read several times. After that, the original expressions were simplified. The purpose of the analysis was to analyse the views of the caring staff caring together, because they work closely as a multi‐professional team (Kesonen et al., 2022). That is why data were analysed as an entity with responses from focus group and individual/couple interviews pooled together. Then simplified expressions were grouped deductively to the matrix of analysis based on the FITT framework and in the following categories: (1) Individual‐task Fit, (2) Individual‐technology Fit and (3) Task‐technology Fit (Ammenwerth et al., 2006; Sheehan et al., 2012). Second, the analysis continued inductively. The data contents in each categories that were similar were grouped into broader subcategories, thus summarizing the data (Elo & Kyngäs, 2008; Table 2).

**TABLE 2:** Accessibility of care plan information from patients' previous care setting in the fit between individual, task and technology framework

Category	Subcategory	Class content
Individual‐task Fit	Professional‐specific care plan information in palliative care	Nursing‐specific perspective
		Medical‐specific perspective
		Social‐specific perspective
	Use of time for receiving care plan information	Use of time for browsing HIS
		Use of time for oral information exchange
Individual‐technology Fit	Health informatics competencies	Health informatics skills
		Attitudes towards technology
	Health information system (HIS) usability	HIS learnability
		Dissatisfaction with the use of HIS
Task‐technology Fit	Interoperability between care settings and healthcare providers	Structural factors as a condition for accessing care plan information
		Interoperability of communication technologies
	Lack of interoperability between care settings and healthcare providers	Structural factors as barriers to accessing care plan information
		Lack of interoperability of communication technologies

### 2.7 Rigour

The trustworthiness of the study was considered in terms of credibility, transferability and confirmability (Polit & Beck, 2017). To confirm credibility, participants in the study represented different healthcare professional groups. The results were described so that the readers are able to understand how the analysis was done. The analysis returned several times to the original expressions of the material to ensure consistency with the categories. The data were first analysed by one researcher, and reliability was confirmed by a multidisciplinary team of researchers by increasing the coverage of the analysis. The original expressions presented confirm the credibility of the study (Elo et al., 2014). Quotes were chosen for clarity, rather than seeking to represent the words of each participant. Pseudonyms were used to ensure anonymity. The quotes from the interviews illustrate the complexity of accessibility of care plan information in palliative care unit. Transferability was assessed using the applicability of the study results (Polit & Beck, 2017). The report describes the selection of participants, the background information of the participants and the collection of data as transparently as possible, protecting anonymity. The target group did not evaluate the results of the study separately; however, the consistency of the results with previous research results increases the confirmability.

## 3 FINDINGS

According to the FITT framework, accessibility of care plan information from patients' previous treatment setting consisted of three areas of fit: Individual‐task Fit, Individual‐technology Fit and Task‐technology Fit (Ammenwerth et al., 2006; Sheehan et al., 2012). Areas of fit serve as categories of results under which subcategories and class content related to descriptions of accessibility of care plan information from patients' previous treatment setting have been formed. The contents of the categories are summarized in Table 2.

### 3.1 Individual‐task fit

Accessibility of care plan information from patients' previous care setting from the perspective of Individual‐task Fit includes two categories (Table 2).

#### 3.1.1 Professional‐specific care plan information in palliative care

Professional‐specific care plan information in palliative care was viewed from the nursing, medical and social perspective.

*From a nursing‐specific perspective*, communication from previous treatment setting to palliative care unit had been developed. Nurses usually received an oral report, but access depended on the nurses being able ask for the right things. Previously, information did not even come on paper. Sometimes they expected printouts deemed necessary by the sending treatment setting. Nurses working in palliative care unit viewed care plan information from patient‐oriented perspective. However, the care plan information received included even active rehabilitation. Thus, a ready‐made nursing care plan was never accessible. Nurses were promised a plan with palliative care needs in HIS, and they were trying to get the things they needed into it.

Or we search the papers to find out what they may have considered it necessary to print out.(FGN2)

We look at those things from quite a different perspective(CIN1)

No ready‐made care plan exists. In principle, we never have what you might call a ready‐made care plan like that for them(FGPN1)

*From a medical‐specific perspective*, care plan information was rarely received by physicians. If it was, they looked more at the information as a whole and at dates, but not so closely at titles or certain (advance) care plan things. Some patients arriving in the palliative care unit were long‐term, mobile patients who occasionally visited their homes, whereas others came to the palliative care unit unconscious, to spend the last few days of their lives there.

Because we so rarely get it …(CIP1)

In our unit they are not… (something) related to the advance care plan(CIP1)

No, I don't look at the headings that much, I look at dates… things as a whole(P2)

From a social‐specific perspective, social workers needed an overall picture of the patient. For example, information needed by nurses and physicians for a procedure was not sufficient them. Sometimes they received care plan information from another organization by phone about something that had remained unfinished.

Perhaps the information we look at is from different perspectives. It's quite possible that some information is insufficient for me now while being sufficient for some other procedure.(S2)

Well in my opinion, quite rarely; like sometimes you get a phone call from a social worker at the X unit saying that something was left unfinished.(S5)

#### 3.1.2 Use of time for receiving care plan information

Use of time for receiving clan plan information meant use of time for browsing HIS and use of time for oral information *exchange*.

*Use of time for browsing HIS* included time to browse nursing and medical records. A new HIS had taken up a lot of time and resources. From the multi‐professional perspective, social workers needed daily nursing notes during the entire treatment period; therefore, browsing the entire nursing record took time if the patient had been on the ward for a long time. On the other hand, care plan information was quickly found in medical records.

Yes it does! That's true, it's so different and like, complicated, and somehow it really takes a lot of energy and, and time(FGN3)

Browsing through the entire Documentation of care takes time if the patient has been on the ward a long time(S3)

The plan can quickly be found on the Speciality page(S3)

*Use of time for oral information exchange* included in‐depth transfer reports by phone or short face‐to‐face summaries. Nursing staff reported that they had a goal of calling for a transfer report where the patient's situation was discussed thoroughly. Sometimes care plan information was based solely on a phone report. Social workers reported that they did not receive much care plan information directly from the previous treatment setting. Care plan information could already have been reviewed when the patient was admitted to hospital. That is why social workers often relied on brief oral care plan information provided by another professional such as nurse or physician.

Social workers felt that if they had the time, participating in medical rounds in the wards would be very informative and useful.

But nowadays, the goal is always to phone in a transition report and … and sometimes you go through the patient's case quite thoroughly in it.(CIN1)

But from the previous site, not much. Only what the professionals in our ward see, like the physician is quickly able to see that…(S2)

IfI had an enormous amount of extra… or like free time, it would be quite useful to take part in the rounds, because during them, they kind of outline and … so it would be really informative.(S4)

### 3.2 Individual‐technology fit

Accessibility of care plan information from patients' previous care setting from the perspective of Individual‐technology Fit includes two categories (Table 2).

#### 3.2.1 Health informatics competencies

Health informatics competencies included health informatics skills and attitudes towards technology.

*Health informatics skills* were seen as a prerequisite for care plan information access. Professionals were able to access information when they were capable of searching for care plan information in the Nursing Documentation System. Local documentation procedures were not uniform, so professionals had to learn on which page of the electronic medical record the care plan information was documented in each municipality. From the technological point of view, previous experience with the use of the same HIS in primary care made it easier to find information.

Yes, when you know how to dig out from the care plan(FGN4)

It's really quite easily discoverable now if you just realize that you have to look for it.(S2)

But in any case, that you find the information, so to speak(P2)

Attitudes towards technology were related to care plan information access. Positive attitudes contributed to discoverability. Finding care plan information in electronic medical records was not considered difficult. Nursing care plan information was found in the Nursing Documentation System and the electronic nursing discharge summary page of HIS, if necessary. All in all, the respondents felt that nothing could be found in the HIS unless one knew what to look for.

So it's not really that difficult(S3)

I can find nurses' documentations in the continuous documentation of care and when I want, I can see them recorded on the electronic nursing discharge summary page,(S2)

It's not that… well you can't find anything in computer systems if you don't know what you are looking for.(S2)

#### 3.2.2 Health information system usability

Health information system usability meant HIS learnability and dissatisfaction with the use of HIS.

*HIS learnability* was manifested in that the new HIS had been introduced unfinished. It was very different from the HIS previously in use. The new HIS had a complex care plan that was difficult to learn. As a result, nurses were not able to use it. One physician said that as end users, they were actually test users and HIS developers.

Now, the care plan in the HIS is so complicated that we don't know how to use it.(FGN3)

This is such a new and recent system that we are kind of developing and using it in unfinished form(P3)

*Dissatisfaction with the use of HIS* appeared especially in retrieving care plan information. According to professionals, searching for information from various data sources was cumbersome. As a result, all care plan information appeared to be scattered. Professionals had to consider where the necessary care plan information might be found. Some care plan information could be found in their own HIS, some in the regional information system and some in the National Patient Data Repository (“Kanta”). The interface of the National Patient Data Archive was not felt to be of any use. For example, no electronic medical case summaries could be found there (Kanta Pages “Kanta”).

It's pretty complicated to look for information. Yes, but it's the system itself, it's somewhat poorly constructed(FGN3)

All the information is scattered there … Really scattered.(FGN3)

The file view is really lousy, and I don't know if patients have been on wards or somewhere over there in X, I can't find the discharge summaries there.(P2)

### 3.3 Task‐technology fit

Accessibility of care plan information from patients' previous care setting from the perspective of Task‐technology Fit includes two categories (Table 2).

#### 3.3.1 Interoperability between care settings and healthcare providers

Interoperability between care settings and healthcare providers included structural factors as a condition for accessing care plan information and interoperability of communication technologies.

*Structural factors as a condition for accessing care plan information* included issues related to privacy and legislation. Health data protection and confidentiality policies were seen as important prerequisites for accessing care plan information. In accordance with current legislation, patients were asked for permission to view their information. With the patient's permission, it was possible to view documentations made in primary care with the use of the regional information system.

Well of course you should ask the patient's permission, like “is it OK if I use this regional view service that we have”.(S1)

That way I can, of course, look at some documentations, if the patient is, like, willing to consent.(S1)

So we can get documentations from the home municipality there …(S1)

Interoperability of communication technologies allowed care plan information from the previous treatment setting to be accessed. The extent to which the information was seen depended on whether the same or a different patient HIS was in use. Patient's previous care plan and daily progress notes were seen if the sending treatment setting used the same HIS. In the case of different HIS, physicians were able to see an electronic nursing discharge summary in the National Patient Data Repository (“Kanta”). Correspondingly, nurses could see physicians' documentations there.

So of course, depending on whether we are using the same data system or different patient information systems, how much of the patient's information we are able to see(CIN1)

You see the old care plan and the daily nursing documentations and such. So it's quite another level of information you get.(CIN1)

So in a way it's visible in Kanta [data repository], because the daily nursing documentations are not visible.(P1)

But of course you can seen at least physicians' notes through the patient information file(CIN1)

#### 3.3.2 Lack of interoperability between care settings and healthcare providers

Lack of interoperability between care settings and healthcare providers meant structural factors as barriers to accessing care plan information and lack of interoperability of communication technologies.

*Structural factors as barriers to accessing care plan information* meant legal issues which prevented seeing all the information needed. Social and health information was not on the same screen of the HIS because municipal social and health services complied with different legislation. The separate personal data registers of different organizations made it difficult to access the data. For example, healthcare professionals working in hospital did not see any documentations of municipal social work. Vice versa, it was difficult to obtain data on the chemotherapy and follow‐up of patients with cancer in home care. According to Finnish law, nursing care plan and daily progress notes were not visible in the National Patient Data Repository (“Kanta”). Thus, the lack of interoperability of patient record registers can be a threat to patient safety. The care plan that accompanied the patient was not realized, but was seen as necessary because the patients were also treated in home care.

… because the documentations made by the municipal social services are not visible to us.(S1)

So it has really made things more complicated as on the oncology side, cytostatic drugs are recorded in the Nursing Documentation System, and the texts as well. And the hospital at home enters documentation in the Nursing Documentation System, but the systems don't communicate with each other.(P1)

But the patient's information includes a documentation of care page that is not transferred to the Kanta service [data repository], for example(S1)

But a care plan that accompanies the patient and is transferred from one institution or site to another, and also to home care – we still don't have that, and it won't happen.(FGPN1)

*Lack of interoperability of communication technologies* meant that patient's care plan information was not available because it was in a separate HIS. They were in different databases that did not discuss with each other and information was not transmitted from another HIS. For example, nursing documentations were poorly seen in organizations using a different HIS. A patient coming to palliative care unit did not have a care plan unless the patient had previously been treated there.

But we use different databases, for example, in municipal hospitals and University Hospital, so they are not available, at least not like that(S4)

But if they come via another system, these nursing care documentations, but the documentation of nursing care is really poorly accessible.(FGN2)

If we get a patient from outside our hospital district, from University Hospital, for example, well in that case we have no care plan on the patient if the patient is not known to us and has not been here before.(FGPN1)

## 4 DISCUSSION

The aim of this study was to describe accessibility of care plan information from patients' previous treatment setting in the palliative care unit. Many challenges from the socio‐technical perspective raised. On the other hand, the results provide solutions that would improve healthcare professionals' access to care plan information in palliative care units.

In this study, *Individual‐task Fit* was described in relation to *professional‐specific care plan information in palliative care* and *use of time for receiving care plan information*. Nurses, physicians and social workers viewed care plan information access and information from their own specific professional perspective, but also collectively from a holistic, patient‐oriented palliative care perspective. However, the results show that care plan information from the patient's previous treatment site did not reflect the patient's overall condition, as recommended by the American Medical Association (2016). This is line with a previous study finding that different professional groups have different information needs (Haavisto et al., 2020) which differ between information producer and recipients (Østensen et al., 2020). Correspondingly, in Denmark, the lack of individualized care plan was the most common barrier for ideal palliative care (Uitdehaag et al., 2018). Professionals accessed care plan information in written form, orally or not at all. The use of multiple information sources was time‐consuming and created challenges for data integration, especially for social workers whose access to care plan information often depends on oral information received from another professional group. This finding is supported by previous research where care plan information widely varied as patients moved from one setting to another (Dykes et al., 2014). Efficient information flow is important because according to Fliedner et al. (2021), well‐made care plans can ease patients' transition between care settings. Payne et al. (2019) highlight the need to promote health information exchange rather than gathering new information. In Australia, a coordinated care approach with a supportive care plan programme has improved communication of treatment goals and facilitated integration of palliative care (Hopkins et al., 2017).

In this study, *Individual‐technology Fit* was described in relation to *health informatics competencies* and *HIS usability*. Accessing care plan information was related to finding it in HIS. Health informatics skills, previous user experiences and positive attitudes contributed to finding care plan information. On the other hand, unfriendly HIS not supporting in daily professional practice was a challenge. Today, everyone uses Google and other search engines at home, but there is no equivalent in HIS. Users' perception of teething troubles is a typical characteristic of HIS. Recently, in their study, Taggart et al. (2021) observed difficulties in engaging professionals in integrated care when no two‐way electronic care plans were available. As a solution, they suggested that when introducing a new technology, it is important that web‐based services to manage care plans should work as seamlessly as possible. It is already known that increasing the use of HIS to access care plan information will fundamentally change professionals' work processes (Whetton, 2005). Learning something new can be challenging, but it is the professionals' responsibility to maintain their professional skills and train or practice (American Medical Association, 2016).

In this study, *Task‐technology Fit* was described in relation to *interoperability between care settings and healthcare providers* and *lack of interoperability between care settings and healthcare providers*. The main concerns raised in these areas were structural factors, such as health data protection and current legislation as barriers. There was a danger of fragmentation of care in situations where HISs did not communicate with each other. In practice, this could lead to inefficiencies, duplication and possibly, increased costs. The situation may be such that treatment‐relevant information about the care plan is not available when needed. Interoperability of data and HIS is required for the smooth use and sharing of care plan information. This is challenging because each organization, unit and professional has their own work processes. The professionals in this study raised the need for a care plan that accompanies the patient from one institution to another. Perhaps an old‐fashioned paper patient file should be introduced that accompanies the patient and in which treatment units print their most important documents. Such an approach would also meet the statutory record‐keeping requirements better than a mere oral report. This is at least until a handy and ubiquitous electronic folder is available. On the other hand, the challenge may be that the information structurally recorded in the HIS may not be printable in an easily readable form on paper to meet the requirements of the recipient of the information. Previously, in their study in the USA, Dykes et al. (2014) noticed that the use of patient‐centred longitudinal care plan supporting care transition was suboptimal. Later, in their study, Payne et al. (2019) compared health information exchange in six countries. They noticed the importance of health information exchange and common concerns about patient confidentiality and data security despite the contextuality of HIS adoption levels and HIS infrastructure. Recently, Sockolow et al. (2020) presented the need of data standards. Documenting standards, such as Continuity of Care Document, organize information that can support the transition of care between organizations (HL7, 2022). Although the evidence is limited, the use of standardized terminologies has potential to enable interoperability across settings (Fennelly et al., 2021).

### 4.1 Strengths and limitations

This study had a multidisciplinary, and especially, nursing‐oriented focus can be considered as the strength of this study. The majority of the previous care planning literature is disease‐oriented or medically focused (Keenan et al., 2008). It can also be considered a strength that the study areas cover three hospital districts. In addition, all but one of the interviews with the study participants were conducted face to face, allowing for visual cues and increasing the reliability of the study (Polit & Beck, 2017).

In turn, it can be considered as a limitation that the interviewees were predominantly nurses, so the views of other professional groups may be less well represented in these results. In turn, this is understandable because most healthcare professionals are nurses. Simultaneous transcribing and preliminary analysis of the data as the interviews progressed to assess saturation was challenging because four researchers collected data in three different geographical areas. The FITT framework (Ammenwerth et al., 2006; Sheehan et al., 2012) used as the framework for analysis kept multiple and complex socio‐technical issues “in view” as they interrelated with each other.

## 5 CONCLUSIONS

In this study, care plan information from previous treatment setting was not sufficiently accessible in the palliative care unit. Multiple interconnections of accessibility of care plan information between different areas of fit were identified. In the area of Individual‐task Fit, care plan information was viewed from the perspective of own professional group, and it took time for receiving information from the continually updated HIS. In the area of Individual‐technology Fit, professionals appeared to have good health informatics skills, but HISs were inappropriate. In the area of Task‐technology Fit, professionals saw sharing care plan information between different organizations and professionals as important. However, they were frustrated by dysfunctional HIS. The data were located in different HISs that did not communicate with each other. This study provided evidence for the changes needed in the socio‐technical system to improve the accessibility of care plan information in the palliative care unit. For example, there is a need to develop an electronic care plan template that accompanies the patient from one institution to another. The HIS should be reviewed as a whole from a holistic and patient‐oriented perspective to enable accessibility of care plan information. The results can be used in the development of data contents, data structures, usability and interoperability of HIS in palliative care. Further research might investigate the quality of care plan information coming from the previous treatment setting to the palliative care unit.

## AUTHOR CONTRIBUTIONS

All authors have agreed on the final version and meet at least one of the following criteria [recommended by the ICMJE (http://www.icmje.org/recommendations/)]:

- substantial contributions to conception and design, acquisition of data or analysis and interpretation of data;
- drafting the article or revising it critically for important intellectual content.

## CONFLICT OF INTEREST

No conflict of interest has been declared by the authors.

## Supporting information


Appendix S1

## Data Availability

The data are not publicly available due to privacy or ethical restrictions.
